# PD-L1/pS6 in Circulating Tumor Cells (CTCs) during Osimertinib Treatment in Patients with Non-Small Cell Lung Cancer (NSCLC)

**DOI:** 10.3390/biomedicines10081893

**Published:** 2022-08-05

**Authors:** Evangelia Pantazaka, Aliki Ntzifa, Argyro Roumeliotou, Evi Lianidou, Vassilis Georgoulias, Athanasios Kotsakis, Galatea Kallergi

**Affiliations:** 1 Laboratory of Biochemistry/Metastatic Signaling, Section of Genetics, Cell Biology and Development, Department of Biology, University of Patras, 26504 Patras, Greece; 2 Analysis of Circulating Tumor Cells Lab, Lab of Analytical Chemistry, Department of Chemistry, National and Kapodistrian University of Athens, 15771 Athens, Greece; 3 Hellenic Oncology Research Group, 11526 Athens, Greece; 4 Department of Medical Oncology, University General Hospital of Larisa, 41334 Larisa, Greece

**Keywords:** circulating tumor cells, non-small cell lung cancer, Osimertinib, PD-L1, pS6

## Abstract

The PD-1/PD-L1 axis provides CTCs an escape route from the immune system. Phosphorylation of the ribosomal protein S6 is implicated in the same pathway, following mTOR activation. The aim of the study was to investigate the expression of PD-L1 and pS6 in CTCs from NSCLC patients under Osimertinib treatment at a single cell level. CTCs were isolated using ISET from NSCLC patients’ blood [37 at baseline, 25 after the 1st cycle, and 23 at the end of treatment (EOT)]. Staining was performed using immunofluorescence. Cytokeratin-positive (CK^+^) CTCs were detected in 62% of patients. CK^+^PD-L1^+^CD45^−^ and CK^+^pS6^+^ phenotypes were detected in 38% and 41% of the patients at baseline, in 28% and 32% after 1st cycle, and in 30% and 35% at EOT, respectively. Spearman’s analysis revealed statistically significant correlations between PD-L1 and pS6 phenotypes at all time points. Survival analysis revealed that CK^+^pS6^+^ (*p* = 0.003) and CK^low^pS6^+^ (*p* = 0.021) phenotypes after 1st cycle were related to significantly decreased one-year progression-free survival (PFS_12m_) and PFS, respectively. CK^+^PD-L1^+^CD45^−^phenotype at baseline and after 1st cycle showed a trend for decreased PFS_12m_. Increased expression of PD-L1/pS6 in CTCs of Osimertinib-treated NSCLC patients implies the activation of the corresponding pathway, which is potentially associated with poor clinical outcomes.

## 1. Introduction

Non-small cell lung cancer (NSCLC) is the most commonly diagnosed type of lung cancer and, together with small-cell lung cancer, is the leading cause of cancer-related mortality in the world. Circulating tumor cells (CTCs) have been identified as crucial players in the progression of numerous types of cancer, including lung cancer [1]. CTCs, after being dislodged from the primary or metastatic tumor, have to survive in the bloodstream and eventually get transferred into distant organs, where they create metastatic loci [1]. Rarity and heterogeneity are among the hallmarks of CTCs [2,3]. Nonetheless, CTCs’ detection and enumeration have been proven to be a useful prognostic tool for both early and advanced NSCLC, with even one CTC being an independent risk factor for recurrence and death [4]. We have recently showcased the complementarity of CTC and ctDNA analysis in NSCLC patients treated with Osimertinib, whereby both are decreased after one cycle of treatment and increased at the end of treatment (EOT), which coincided with the progression of the disease [5]. Osimertinib is a third-generation epidermal growth factor receptor (EGFR) tyrosine kinase inhibitor (TKI) [6]. *EGFR* mutations in NSCLC occur mainly in exons 18–21. The sensitizing/activating mutations (exon 19 deletions and point mutation L858R located in exon 21) are the most common, and NSCLC patients bearing these mutations usually respond well to EGFR TKI therapy [7,8]. The T790M and C797S point mutations in exon 20 have been linked with resistance to first-generation EGFR TKI therapies. Point mutations G719X, S768I, and L861Q are less frequent [7,8]. A previous study of our group in NSCLC patients treated with Osimertinib (a subset of these patients has been evaluated herein) identified the aforementioned *EGFR* mutations (with the exception of L861Q) in 62% of patients [5]. Osimertinib is effective against mutations L858R, exon 19 deletions, and T790M [9].

In the era of precision and individualized treatment, immunotherapy has created quite a sensation establishing itself as the standard of care in NSCLC. Anti-programmed cell death ligand-1 (PD-L1)/programmed cell death protein-1 (PD-1)-based therapies (such as atezolizumab, a humanized monoclonal antibody against PD-L1, and pembrolizumab, a humanized IgG4 monoclonal antibody against PD-1) have certainly improved patients’ lives in recent years. PD-L1 is expressed in lymphoid and non-lymphoid tissues, as well as tumor cells, while PD-L1’s receptor PD-1 is expressed on B, T, and myeloid cells. Interaction between PD-L1 on a tumor cell and PD-1 on an activated T cell can halt expected immune responses, which would eliminate the target, namely the tumor cell [10]. CTCs can manipulate the PD-L1/PD-1 interaction in their favor, avoid the strict surveillance of the immune system and hence manage to survive, metastasize and thrive. Thus, PD-L1 has been extensively studied in NSCLC [10].

Expression of PD-L1 on CTCs has been reported in various types of cancer, including NSCLC. Expression of PD-L1 varies among studies depending on the detection method (label-dependent vs. -independent) and patients’ treatment. In terms of the label-independent isolation methods, PD-L1^+^ CTCs isolated by Parsortix were detected in 20% of advanced NSCLC patients before anti-PD-1 treatment [11], in 35% of advanced NSCLC patients undergoing pembrolizumab therapy [12] and in 56% of NSCLC patients’ samples [13]. By using the spiral microfluidic technology, 56% of stage IV patients were found to have ≥1 PD-L1^+^ CTCs [14]. Using the MCA system, 73% of stage II-IV patients expressed PD-L1 in their CTCs [3]. Finally, among 22 metastatic NSCLC patients receiving anti-PD-1/PD-L1 treatment, 97% of the cytokeratin (CK)^+^ patients had ≥1 PD-L1^+^ CTCs, which were isolated by vortex HT chip [15]. All these different percentages of PD-L1 expression signify the complexity associated with the detection and characterization of CTCs. Regarding the ISET system, which was also used in our study, 54% of stage II-IV NSCLC patients had PD-L1^+^ CTCs [16]. An earlier study in advanced stage III-IV NSCLC patients had identified only 8% of the patients with >1% PD-L1^+^ CTCs [17]. We have previously shown that PD-L1^+^ CTCs could be detected in patients with metastatic NSCLC before (37%) and after (46%) the third cycle of chemotherapy [18]. Recently, *PD-L1* gene expression has been reported to be increased in NSCLC patients after a cycle of Osimertinib treatment (28%) and at EOT (35%), compared to baseline (10%) [19].

In lung cancer, PD-L1 has been recently found to be regulated by mTOR, which lies further down the EGFR pathway [20]. A downstream event following activation of mTOR is the phosphorylation of the ribosomal protein S6 (pS6). pS6, as a component of the 40S ribosomal subunit, is involved in protein translation, but also cell proliferation [21,22]. pS6 expression has been found elevated in NSCLC, and when down-regulated, it inhibited NSCLC growth [23]. Overexpression of pS6 in lung adenocarcinoma has been associated with metastatic tumors and statistically significant shorter time to metastasis [24]. Evaluation of PD-L1 and pS6 (as an indicator of mTOR activation) in human lung tissue microarrays of adenocarcinomas and squamous cell carcinomas revealed a statistically significant correlation between the two markers, with 40% of lung tumors expressing both proteins. A total of 90% of tumors expressing PD-L1 also expressed pS6, while tumors negative for pS6 were also negative for PD-L1 [25].

The goal of the study was to investigate the expression of PD-L1 and pS6 in CTCs isolated from NSCLC patients treated with Osimertinib at baseline, after the first cycle of treatment, and at EOT.

## 2. Materials and Methods

### 2.1. Patients and Clinical Samples

Forty-two patients [11 men and 31 women; median age 67 years old (range: 43–87)] with NSCLC were enrolled in the study. All patients had adenocarcinoma. All patients filled out a written informed consent, which was approved by the Ethics Committees and the Institutional Review Boards of the participating hospitals, the National Ethic Committee (no: 35/00-03/16), the National Drug Organization (no: IS 28/16) and registered in the clinicaltrials.gov platform (number: NCT02771314, registration date: 13 May 2016) and EudraCT (number: 2016-001335-12, registration date: 13 April 2016). Peripheral blood samples (20 mL in EDTA tubes, after the first 5 mL were discarded to avoid contamination from skin epithelial cells) were collected at different time points; samples from 37 patients were obtained at baseline (B, prior to Osimertinib treatment), samples from 25 patients were obtained after one cycle of treatment (P 1st), and samples from 23 patients were obtained at EOT. 

### 2.2. Cell Culture and Cytospin Preparation

H1299 and A549 (obtained from ATCC) were used for the evaluation of PD-L1 and pS6 expression, respectively [18]. Both were cultured in DMEM Glutamax (Thermo Fisher Scientific, Waltham, MA, USA) supplemented with 10% fetal bovine serum (FBS; PAN-Biotech, Aidenbach, Germany), and 50 U/mL penicillin/50 μg/mL streptomycin (Thermo Fisher Scientific, MA, USA). MCF7 (obtained from ATCC) were also used for the evaluation of pS6 expression [26,27]. MCF7 were cultured in DMEM Glutamax (Thermo Fisher Scientific, MA, USA) supplemented with 10% fetal bovine serum (FBS; PAN-Biotech, Germany), 0.01 mg/mL human recombinant insulin, and 50 U/mL penicillin/50 μg/mL streptomycin (Thermo Fisher Scientific, MA, USA). Cells were maintained at 37 °C in 5% CO_2_. Sub-cultivation was performed with 0.25% trypsin-EDTA (Thermo Fisher Scientific, MA, USA). Cancer cells were spiked into normal donors’ peripheral blood mononuclear cells (PBMCs; 1000 cancer cells/100,000 PBMCs) to mimic the CTC microenvironment in the control samples. Cell lines were used as positive and negative controls for antibodies’ specificity and sensitivity.

### 2.3. Isolation of CTCs

CTCs were isolated from 10 mL of peripheral blood using the ISET system (Rarecells, Paris, France). Dilution and filtration of the samples were as per the manufacturer’s instructions, also referenced in previous work [18]. Membranes were stored at −20 °C until immunofluorescence staining.

### 2.4. Immunofluorescence and Confocal Imaging

ISET filters were washed with PBS, permeabilized with 0.5% Triton-X-100 in PBS for 10 min, and blocked with 10% FBS in PBS for 1 h at 20 °C. For the combination of CK/PD-L1/CD45, filters were first incubated with the goat anti-PD-L1 antibody (1:100 in 1% FBS/PBS, Novus Biologicals, Littleton, CO, USA) for 1 h at 20 °C, washed three times and incubated with an anti-goat Alexa Fluor 488 secondary antibody (1:500; Life Technologies, Carlsbad, CA, USA) for 45 min at 20 °C. Filters were then incubated with a cocktail of antibodies [1:100 mouse anti-CK7 (Invitrogen, Waltham, MA, USA) and 1:70 mouse A45 B/B3 anti-human CK (Amgen, Thousand Oaks, CA, USA) in 1% FBS/PBS] for 1 h at 20 °C, washed three times with PBS and incubated with a donkey anti-mouse Alexa Fluor 555 secondary antibody (1:500; Life Technologies, United States) for 45 min at 20 °C. Filters were washed three times, and finally, filters were incubated with the mouse anti-CD45 antibody conjugated with Alexa 647 (1:70 in 1% FBS/PBS; Novus Biologicals) for 1 h at 20 °C. Filters were washed three times and mounted on Prolong antifade medium containing DAPI. Slides were stored at −20 °C until imaging.

The process was similar for the CK/pS6 staining. In brief, cells were first stained for CK as described above, and then filters were incubated with a rabbit anti-pS6 antibody, specific for the phosphorylation of S6 at S235/236 (1:100 in 1% FBS/PBS; Cell Signaling, Danvers, MA, USA) for 1 h at 20 °C, followed by three washes and incubation with an anti-rabbit Alexa 488 (1:600 in 1% FBS/PBS) for 45 min. Cytospins of PBMCs from healthy donors spiked with H1299 (for PD-L1), and MCF7 or A549 (for pS6) were used as controls (Figure S1). Positive controls were stained with all primary and secondary antibodies, while negative controls for each antibody lacked the primary antibody of interest, while they were incubated with the corresponding secondary anti-IgG. Fixation/permeabilization for cytospins was performed either in ice-cold acetone/methanol (9:1) for 20 min (for PD-L1 experiments) or in 3.7% formaldehyde for 10 min, followed by three washes and a 10 min incubation in 0.5% Triton-X-100 (for pS6). Slides were left overnight in blocking solution (2.5–5% FBS).

Imaging was performed with a Leica TCS SP8 confocal microscope with a ×40 oil-immersion objective. Controls established that there was no bleed-through for none of the combinations examined. Criteria for CTCs’ identification included a high nuclear to cytoplasmic ratio, the presence of double nuclei, and the dimension of the long diameter of the nucleus to be 10 μm or longer [16,28]. Overview of fields with multiple cells is provided in Figure S2. For the characterization of the various phenotypes, fluorescence after staining for CK was characterized as “normal” (CK^+^) and low (CK^low^) compared to the background expression in the patients’ PBMCs. CK^low^ expression was slightly over background fluorescence. These are considered CTCs under epithelial-to-mesenchymal transition (EMT) since they satisfy the cytomorphological criteria of cancer cells, according to Meng et al. (2004). Representative images of CK^+^- and CK^low^-expressing CTCs are presented in Figure S3. Panels of (CK^+^ vs. CK^low^) cells belong to the same patient, so the difference in fluorescence can be comparable.

### 2.5. Statistical Analysis

Analysis has been performed in terms of total patients for each time point, as well as in terms of the patients who actually had CTCs at each time point, referred to as CK^+^ patients. The latter analysis provides an indication of the heterogeneity among patients who harbor CTCs.

Non-parametric tests were used to analyze the relationships between phenotypes (Chi-square and Kruskal–Wallis tests were used to estimate differences between 2 and 3 groups, respectively). Correlation between phenotypes was calculated using Spearman’s coefficient. Kaplan–Meier analysis was used to estimate progression-free survival (PFS) and overall survival (OS). *p* < 0.05 was considered significant. Analysis was performed using the SPSS software (version 26, IBM, Armonk, NY, USA).

## 3. Results

### 3.1. Detection of CTCs in NSCLC Patients

Based on the CK staining, CTCs could be detected in 62% of total patients (26 out of 42). More specifically, concentrating on the different time points, CK^+^ cells could be found in 51% of patients (19 out of 37) before any treatment at baseline, in 44% of patients (11 out of 25) after the first cycle of Osimertinib treatment (post first cycle), and in 35% of patients (8 out of 23) at EOT. Interestingly, there was a slight decrease in CTCs after one cycle of treatment and at EOT, suggesting that Osimertinib treatment might have slightly affected CTCs count.

### 3.2. Detection of PD-L1^+^ CTCs in NSCLC Patients and Identification of Different Phenotypes

**Figure 1:**
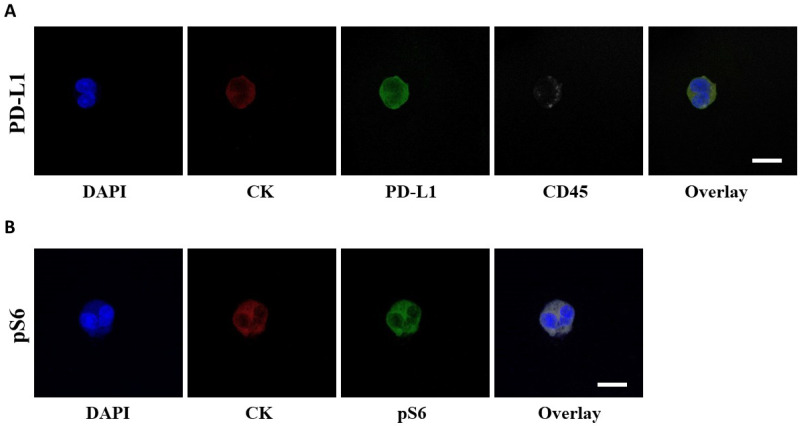
PD-L1 and pS6 expression in NSCLC patients’ CTCs. (**A**) CTCs stained with CK (red), PD-L1 (green) and CD45 (grey). Nuclei (blue) were stained with DAPI. (**B**) CTCs stained with CK (red), pS6 (green) and nuclei (blue). The overlay of all images is also presented. Scale bars = 10 μm.

The presence of PD-L1^+^ CTCs was evaluated by immunofluorescence (Figure 1A). The rate of detection of PD-L1^+^ CTCs in total patients was 38% (14 out of 37) at baseline; it decreased to 28% (7 out of 25) after the first cycle of treatment and marginally increased to 30% (7 out of 23) at EOT.

**Figure 2:**
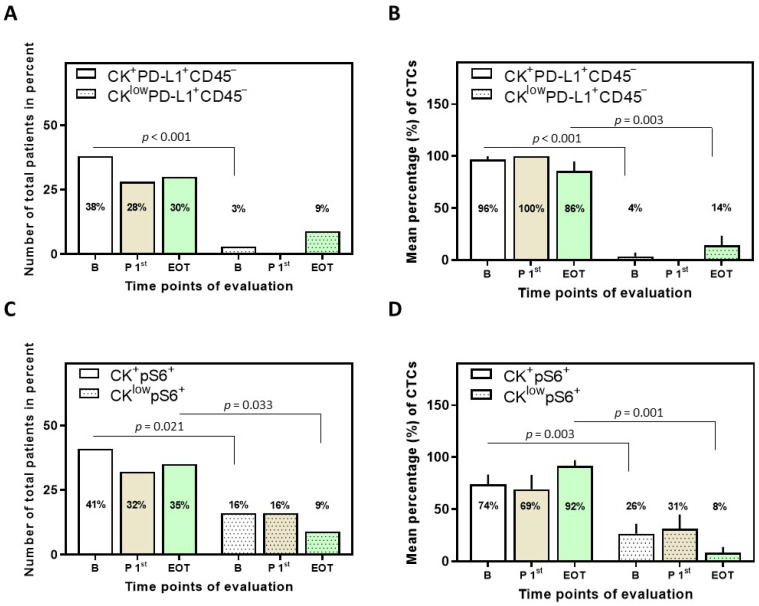
Phenotypes of PD-L1 and pS6 identified in NSCLC patients and their CTCs. (**A**) Percentage of total patients with the identified phenotypes of PD-L1 (*p* < 0.001 among the phenotypes at baseline) and (**B**) mean percentage of CTCs with the identified phenotypes of PD-L1 (*p* < 0.001 and *p* = 0.003 among the phenotypes at baseline and EOT, respectively). (**C**) Percentage of total patients with the identified phenotypes of pS6 (*p* = 0.021 and *p* = 0.033 among the phenotypes at baseline and EOT, respectively) and (**D**) mean percentage of CTCs with the identified phenotypes of pS6 (*p* = 0.003 and *p* = 0.001 among the phenotypes at baseline and EOT, respectively). Results [in (**B**,**D**)] are expressed as mean ± SEM. B (white bars), baseline; P 1st (purple bars), post 1st cycle; EOT (green bars), end of treatment.

CK^+^PD-L1^+^CD45^−^ and CK^low^PD-L1^+^CD45^−^ were the two phenotypes observed among total patients (Figure 2). The CK^+^PD-L1^+^CD45^−^ phenotype was found in 38% (14 out of 37) of total patients at baseline, in 28% (7 out of 25) after the first cycle of treatment, and in 30% (7 out of 23) at EOT. Interestingly, χ^2^ analysis revealed a statistically significant difference in the CK^+^PD-L1^+^CD45^−^ phenotype between baseline and after a cycle of treatment, suggesting that Osimertinib did indeed have an effect on the CTCs expressing the phenotype (*p* = 0.011). The CK^low^PD-L1^+^CD45^−^ phenotype was found in 3% (1 out of 37) of total patients at baseline and in 9% (2 out of 23) at EOT (Figure 2A).

Among the CK^+^ patients, the frequency of the CK^+^PD-L1^+^CD45^−^ phenotype was 100% at baseline (14 out of 14), after the 1st cycle (7 out of 7), and at EOT (7 out of 7). The CK^low^PD-L1^+^CD45^−^ phenotype was seen at baseline in 7% (1 out of 14) and at EOT in 29% (2 out of 7) of CK^+^ patients (Figure S4A).

Examination of the mean percentage of the total isolated CTCs per patient revealed that the CK^+^PD-L1^+^CD45^−^ phenotype was the most prevalent; 96 ± 4%, 100%, and 86 ± 9% for the different time points (Figure 2B). These results suggest that PD-L1 is highly expressed in CTCs of NSCLC patients before, during, and after Osimertinib treatment, with a slight decrease at EOT, albeit not statistically significant. Conversely, the CK^low^PD-L1^+^CD45^−^ phenotype was increased (again without statistical significance) at EOT (14 ± 9%) compared to baseline (4 ± 4%) and post first cycle (0%). Statistical significance was observed at baseline and EOT among the two phenotypes (Figure 2B).

### 3.3. Detection of pS6^+^ CTCs in NSCLC Patients and Identification of Different Phenotypes

The presence of pS6^+^ CTCs was evaluated by immunofluorescence (Figure 1B). The rate of detection of CTCs in this staining in total patients was 51% (19 out of 37) at baseline; it decreased to 44% (11 out of 25) after the first cycle of treatment and further decreased to 35% (8 out of 23) at EOT.

CK^+^pS6^+^ and CK^low^pS6^+^ were the two phenotypes observed among total patients (Figure 2C). The CK^+^pS6^+^ phenotype was found in 41% (15 out of 37) of total patients at baseline, 32% (8 out of 25) after the first cycle of treatment, and 35% (8 out of 23) at EOT. The CK^low^pS6^+^ phenotype was found in 16% (6 out of 37) of total patients at baseline, 16% (4 out of 25) after the first cycle of treatment, and in 9% (2 out of 23) at EOT (Figure 2C).

Among the CK^+^ patients, the frequency of the CK^+^pS6^+^ phenotype was 79% at baseline (15 out of 19), 73% (8 out of 11) post 1st cycle, and all (8 out of 8) at EOT. The CK^low^pS6^+^ phenotype was seen at baseline in 32% (6 out of 19) of CK^+^ patients, post 1st cycle in 36% (4 out of 11), and at EOT in 25% (2 out of 8) of CK^+^ patients (Figure S4B).

Examination of the mean percentage of the total isolated CTCs per patient further revealed that the CK^+^pS6^+^ phenotype was the most prevalent; 74 ± 10%, 69 ± 14% and 92 ± 5% for the different time points (Figure 2D). These results suggest that pS6 is highly expressed in CTCs of NSCLC patients before, during, and after Osimertinib treatment, with an increase at EOT, albeit not statistically significant.

**Figure 3:**
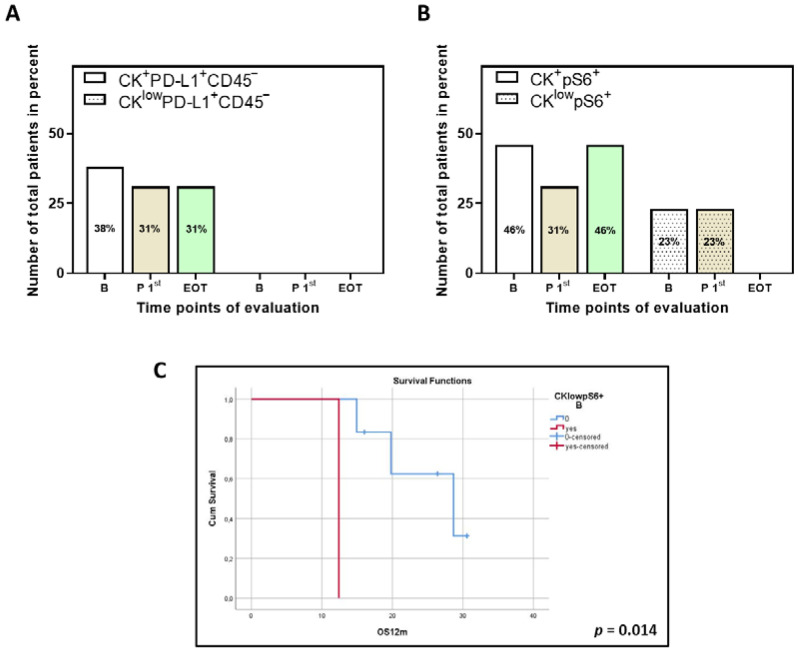
Phenotypes of PD-L1 and pS6 identified in thirteen NSCLC patients for which samples for all time points are available. Percentage of total patients with the identified phenotypes of (**A**) PD-L1 and (**B**) pS6. B (white bars), baseline; P 1st (purple bars), post 1st cycle; EOT (green bars), end of treatment. (**C**) Kaplan–Meier analysis of 1-year OS (OS_12m_) for pS6 for the thirteen patients with samples at all time points. OS_12m_ according to the presence or absence of the phenotype CK^low^pS6^+^ (B, *p* = 0.014). B, baseline.

Additional analysis was also performed for thirteen patients for which samples at the three time points of interest were available (Figure 3). The predominance of the phenotypes CK^+^PD-L1^+^CD45^−^ and CK^+^pS6^+^ was similar in this subset of these patients, as was the trend of abundance in the different time points. The CK^low^PD-L1^+^CD45^−^ phenotype was not detected in this subset, which is not unexpected since its percentage was already low in the initial cohort of patients.

### 3.4. Correlation between PD-L1 and pS6 Expression in NSCLC Patients’ CTCs

Interesting correlations between the two markers and their corresponding phenotypes were detected at the three different time points of interest following Spearman analysis (Table 1).

More specifically, a correlation was observed between the detection of the two predominant phenotypes CK^+^PD-L1^+^CD45^−^and CK^+^pS6^+^ at all time points (*p* < 0.001 at baseline, *p* = 0.004 post 1st cycle and *p* < 0.001 at EOT). In addition, CK^+^PD-L1^+^CD45^−^ and CK^low^pS6^+^ also correlated at all time points (*p* = 0.035 at baseline, *p* = 0.010 post 1st cycle and *p* = 0.039 at EOT). For the phenotypes CK^low^PD-L1^+^CD45^−^ and CK^low^pS6^+^ correlation was seen at baseline (*p* = 0.011) and at EOT (*p* = 0.030; Table 1).

These results suggest that PD-L1 and pS6 potentially coexist in the same signal transduction cascade, related to the mTOR pathway, showcased via the phosphorylation of the ribosomal S6 protein.

### 3.5. Clinical Outcome Based on the Presence of PD-L1^+^ and pS6^+^ CTCs

**Figure 4:**
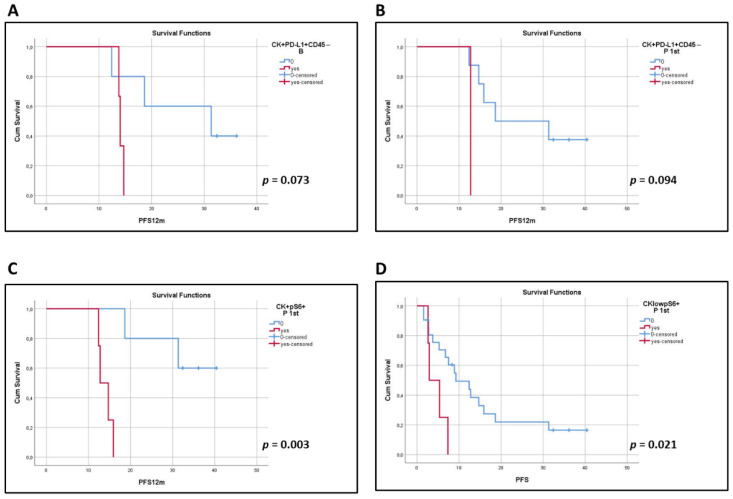
Kaplan–Meier analysis of 1-year PFS (PFS_12m_) and PFS for PD-L1 and pS6. PFS_12m_ according to the presence or absence of the identified PD-L1 and pS6 phenotypes; (**A**) CK^+^PD-L1^+^CD45^−^ (B, *p* = 0.073), (**B**) CK^+^PD-L1^+^CD45^−^ (P 1st, *p* = 0.094) and (**C**) CK^+^pS6^+^ (P 1st, *p* = 0.003). (**D**) PFS for the presence or absence of CK^low^pS6^+^ (P 1st, *p* = 0.021). B, baseline; P 1st, post 1st cycle.

Clinical data were available for 42 patients. Patients’ characteristics, the median number of treatment cycles and the range of EOT occurrence are presented in Table 2. Among total patients, the phenotype CK^+^PD-L1^+^CD45^−^ at baseline (log-rank *p* = 0.073) and after the 1st cycle of treatment (log-rank *p* = 0.094) showed a trend for shorter PFS_12m_ (Figure 4A,B).

In terms of pS6 expression, the CK^+^pS6^+^ and CK^low^pS6^+^ phenotypes after the 1st cycle of treatment were correlated with shorter PFS_12m_ (log-rank *p* = 0.003; Figure 4C) and PFS (log-rank *p* = 0.021, HR = 2.09, 95% C.I. = 1.02–4.28; Figure 4D), respectively. Interestingly, these results are supported by data provided by KMplot (https://kmplot.com/analysis, accessed on 3 March 2022), an online tool, whereby high expression of the *pS6* gene has been indicated to have a shorter First Progression Interval (log-rank *p* = 2.1 10^−5^, HR = 1.52; Figure S5A) and OS (log-rank *p* = 1.5 10^−8^, HR = 1.45; Figure S5B) in adenocarcinomas and squamous cell carcinomas of all stages and grades [29].

Finally, survival analysis performed on the thirteen patients for which samples at the three time points of interest were available showed that the phenotype CK^low^pS6^+^ at baseline was associated with decreased OS_12m_ (log-rank *p* = 0.014; Figure 3C).

## 4. Discussion

The low detection rate of CTCs in NSCLC, which is frequently reported, is attributed to the epithelial-to-mesenchymal transition (EMT) characteristics of tumor cells [1]. However, this has not detracted from the fact that the presence of even one CTC has been correlated with poor prognosis [30]. Detection of CTCs in NSCLC patients has been widely associated with reduced PFS [11,12,30,31,32,33] and OS [12,30,31,32,33]. CTC positivity in NSCLC, whether CTCs have been identified by label-dependent or -independent methods and irrespective of treatment regime, has been reported to range between approx. 43–100%, with the ISET technology showing far better detection ability and recovery [3,11,14,16,17,18,19,30,32,34,35,36,37,38,39]. Therefore, in the current study, ISET technology has been used for the isolation of CTCs. In a previous study of our group, CK^+^ CTCs were identified by IF on ISET filters from 30 chemo-naïve stage IV NSCLC patients at baseline, before front-line chemotherapy (57% of patients), and after the third cycle of treatment (73%) [18]. In addition, in another of our studies, 69% of NSCLC patients treated with Osimertinib had CTCs isolated by ISET [5]. In the current study, 62% (26 out of 42) of patients had at least one CTC at some point in the evaluation, which is comparable to Kallergi et al. (2022).

Expression of PD-L1 on CTCs has been reported in various types of cancer, including NSCLC. In our study, two PD-L1 phenotypes were detected, namely CK^+^PD-L1^+^CD45^−^ and CK^low^PD-L1^+^CD45^−^. Of the CK^+^ CTCs, all exhibited the CK^+^PD-L1^+^CD45^−^ phenotype at all time points, suggesting that Osimertinib cannot influence these subclones of CTCs. The same observation was made regarding the effect of adjuvant chemotherapy on PD-L1^+^ CTCs in a previous study of our group [18]. More specifically, PD-L1^+^ CTCs were detected on ISET filters in 27% of patients before and in 46% after the third cycle of chemotherapy [18]. Similarly, in a group of 13 non-metastatic patients, with a 69% overall PD-L1 positivity rate detected via a microfluidic chip, PD-L1^+^ CTCs were found to increase after (chemo)radiation therapy [40]. In a later report, all treatment-naïve NSCLC patients after surgery were also found to have PD-L1^+^ CTCs, isolated by CellSieve [39].

Interestingly, in terms of immune checkpoint inhibitors (ICIs) and, more specifically, nivolumab treatment, reports have been contradictory, with some suggesting decrease in PD-L1^+^ CTCs [37,41] and others reporting an increase [32]. Ikeda et al. (2021) longitudinally evaluated the PD-L1 positivity rate in CTCs, from advanced NSCLC patients isolated by the MCA system, at baseline (82%) and after 4, 8, 12, and 24 weeks, following nivolumab treatment, with rates of 58%, 56%, 62%, and 55%, respectively [41]. In Nicolazzo et al. (2016), while 95% of CK^+^ patients had PD-L1^+^ CTCs at baseline and all had after 3 months of treatment, only 50% ended up having PD-L1^+^ CTCs after 6 months [37]. On the other hand, in another report, 83% of advanced metastatic patients expressed PD-L1 at baseline, while all patients expressed PD-L1 in their ISET-isolated CTCs at the time of progression [32]. This suggested that high PD-L1^+^ CTCs at baseline could be correlated with decreased nivolumab response and resistance to ICI treatment [13,32]. Our high PD-L1 positivity rate resembles that reported by Guibert et al. (2018), with the added advantage of having a higher number of total patients.

Finally, in terms of TKI therapies, *PD-L1* gene expression, using Real-Time PCR, in CTC fractions isolated by Parsortix from NSCLC patients treated with Osimertinib (a subset of the patients was used in this study for single cell analysis) has been shown to be low (10%; 3 out of 30 patients) at baseline, to increase after the first cycle of treatment (28%; 7 out of 25) and further increase at EOT (35%; 9 out of 26) [19]. The trend seen at the mRNA level was different at the protein level at baseline (10% vs. 38%); however, there was concordance after the first cycle of treatment (28% both) and at EOT (35% vs. 30%). Interestingly a subclone with a more mesenchymal phenotype (CK^low^PD-L1^+^CD45^−^) was statistically increased at EOT, implying that the picture is more complicated and informative at the protein level with single cell analysis.

The presence of PD-L1^+^ CTCs in previous reports has either been associated with significantly worse OS [30,42], PFS [40], or disease-free survival (DFS) compared to patients having PD-L1^−^ CTCs [35]. However, in other studies, the presence of PD-L1^+^ CTCs was associated with only a trend for worse PFS [16,30], which was not statistically significant, or even no correlation at all with PFS [11,32,38,43] or OS [11,32,35,38,41]. On two occasions, the presence of PD-L1^+^ CTCs was associated with longer PFS and/or OS [34,41]. In a recent meta-analysis, the existence of PD-L1^+^ CTCs was not associated with PFS or OS; however, in ICI-treated patients, the presence of PD-L1^+^ CTCs predicted better survival compared to patients undergoing other therapies [44]. In our study, the presence of PD-L1 and, more specifically, the phenotype CK^+^PD-L1^+^CD45^−^ at baseline and after one cycle of treatment demonstrated a trend towards lower PFS_12m_ (log-rank *p* = 0.073 and log-rank *p* = 0.094, respectively).

Apart from the PD-L1/PD-1 pathway, the EGFR signaling cascade is of great importance in NSCLC. Activation of the receptor leads to activation of downstream pathways such as RAS/RAF/ERK and PI3K/AKT/mTOR [45]. Both subsequently up-regulate PD-L1. The involvement of mTOR in the regulation of PD-L1 expression has been controversial among different types of cancer [20]. In NSCLC, inhibition of the mTOR pathway has been shown to either enhance [46] or decrease [25,47] PD-L1 expression. mTORC1, one of the two complexes of the Ser/Thr kinase mTOR, is responsible for the activation of the p70 ribosomal protein S6 kinase, which phosphorylates the ribosomal protein S6 [48]. Interestingly, apart from the PI3K/AKT pathway, the RAS/ERK pathway has also been described to activate the phosphorylation of S6 (discussed in [49,50]).

pS6 has been reported to be expressed in approx. 30–80% of primary adenocarcinoma samples and 30–60% of tissue arrays [24,48,50,51]. pS6 expression was significantly higher in stage I NSCLC adenocarcinomas compared to squamous cell carcinomas [52]. In addition, pS6 expression in brain metastatic lung adenocarcinomas was significantly higher compared to primary adenocarcinomas, while expression in normal lungs was considerably low [48]. To the best of our knowledge, this is the first study to evaluate pS6 expression in CTCs. The predominant phenotype which was detected was CK^+^pS6^+^ and was found in 79% of CK^+^ patients at baseline, 73% post 1st cycle, and in all patients at EOT. In addition, the mean percentage of CTCs expressing pS6 slightly decreased after one cycle of treatment (69%) to increase, although without statistical significance, at EOT (92%).

In NSCLC tissues, PD-L1 expression has been shown to negatively correlate with pS6 expression [46], whereas in another study, tumors not expressing pS6 did not express PD-L1 either [25]. In our study, expression of PD-L1 at every time point correlated with that of pS6 (Table 1), indicating that the corresponding pathway downstream of EGFR is activated. Moreover, it is obvious that both PD-L1 and pS6 expression is not affected by Osimertinib treatment, and both are high at EOT. In patients undergoing EGFR TKI treatment, one would expect blockage of EGFR signaling, as well as of both RAS/RAF/ERK and PI3K/AKT pathways. This would ultimately mean a reduction of PD-L1 expression. Interestingly, activation of AKT has been associated with resistance to EGFR TKI in in vitro NSCLC studies (discussed in [49]). This could potentially provide an explanation for the 100% PD-L1 expression at EOT in CK^+^ patients.

In terms of a link between pS6 expression and patients’ clinical outcome, CK^+^pS6^+^ and CK^low^pS6^+^ after the 1st treatment cycle were correlated with shorter PFS_12m_ (log-rank *p* = 0.003) and PFS (log-rank *p* = 0.021), respectively. In addition, in the cohort of patients for which samples for all time points were available, the CK^low^pS6^+^ phenotype at baseline was associated with reduced OS_12m_ (log-rank *p* = 0.014). However, due to the small number of the latter cohort, these results are only indicative of the potential importance of pS6 expression in CTCs for NSCLC patients. These findings are also in line with previous studies showing that overexpression of pS6 in lung adenocarcinoma and tissue microarrays has been associated with a shorter time to metastasis and shorter OS [24,51]. In accordance, based on KMplot (https://kmplot.com/analysis, accessed on 3 March 2022), high expression of the *pS6* gene in primary tumors correlates with shorter OS [29].

Overall, CTCs could be detected in half of the total patients at baseline, with a slight decrease following Osimertinib treatment. Both PD-L1 and pS6 expression in total patients was slightly higher at baseline. CK^+^PD-L1^+^CD45^−^ and CK^+^pS6^+^ were the two predominant phenotypes detected. Correlation among the PD-L1 and pS6 phenotypes further suggests a dependence of the expression of the former by the activation and corresponding pathway of the latter. The presence of the CK^+^pS6^+^ and CK^low^pS6^+^ (after one cycle of treatment) were correlated with shorter PFS_12m_ and PFS. Furthermore, the presence of pS6 in CTCs was also correlated with poorer OS_12m_ in a cohort of thirteen patients (with available all the examined time points), implying an important role of this biomarker for NSCLC patients. A limitation of the current report is the relatively small number of patients enrolled; however, this was a pilot study to evaluate the expression of pS6 and PD-L1 as biomarkers during Osimertinib treatment. These numbers of patients are comparable with other previous studies in the literature [32,39,41]. Therefore, due to the nature of the study and the three different time points, the number of samples that were evaluated (85 per staining) was sufficient to give an initial indication of the clinical significance of these biomarkers. Nevertheless, future studies in a larger group of patients will elucidate the clinical impact of PD-L1 and pS6 as biomarkers in CTCs from NSCLC patients.

## 5. Conclusions

Single-cell analysis revealed that NSCLC patients expressed high levels of PD-L1 and pS6 proteins in their CTCs. pS6 expression was correlated to patients’ clinical outcomes. Osimertinib treatment did not significantly change the number of PD-L1^+^ or pS6^+^ CTCs, implying that this distinct (EGFR–PI3K–AKT–mTOR–S6) pathway is constantly activated in NSCLC patients. Combining treatment with TKI inhibitors such as Osimertinib and ICIs could potentially target more effectively the corresponding pathway and reduce the metastatic dissemination of cancer cells.

## Figures and Tables

**Table 1:** Statistically significant correlations between PD-L1 and pS6 expression among time points of evaluation.

Phenotypes (Time Points)		*p*
CK^+^PD-L1^+^CD45^−^ (B)	CK^+^pS6^+^ (B)	<0.001
	CK^low^pS6^+^ (B)	0.035
CK^low^PD-L1^+^CD45^−^ (B)	CK^low^pS6^+^ (B)	0.011
CK^+^PD-L1^+^CD45^−^ (P 1st)	CK^+^pS6^+^ (P 1st)	0.004
	CK^low^pS6^+^ (P 1st)	0.010
CK^+^PD-L1^+^CD45^−^ (EOT)	CK^+^pS6^+^ (EOT)	<0.001
	CK^low^pS6^+^ (EOT)	0.039
CK^low^PD-L1^+^CD45^−^ (EOT)	CK^low^pS6^+^ (EOT)	0.030

B, baseline; P 1st, post 1st cycle; EOT, end of treatment.

**Table 2:** Patients’ characteristics.

Characteristics	*n* (%)
**Age (years)**	
Median	67
Range	43–87
**Gender**	
Male/Female	11 (26)/31 (74)
**Histological type**	
Adenocarcinoma	42 (100)
**Best response**	
Complete	1 (2)
Partial	12 (29)
Stable disease	16 (38)
Progression disease	13 (31)
**Last treatment cycle**	
Median	9
Range	2–58

## Data Availability

Data presented in the study are available upon request from the corresponding author.

## Supplementary Materials

The following supporting information can be downloaded at: https://www.mdpi.com/article/10.3390/biomedicines10081893/s1. Figure S1: Cytospins of PBMCs from normal donors spiked with (A) H1299 and (B) MCF7 (breast) or A549 (lung) cells and stained for (A) CK (red)/PD-L1 (green)/CD45 (grey) and (B) CK (red)/pS6 (green). Overlays are presented. Scale bars = 10 μm. Figure S2: Representative fields with multiple cells. CTCs are stained for CK (red) and pS6 (green), and nuclei (blue) are visible with DAPI. The overlay of all images is also presented. Scale bars = 10 μm. Figure S3: Representative panels of CK^+^- and CK^low^-expressing cells among the same patients. CTCs are stained for CK (red), and nuclei (blue) are visible with DAPI. Arrows pinpoint the cell of interest. Scale bar = 10 μm. Figure S4: Phenotypes of PD-L1 and pS6 identified in NSCLC patients. Percentage of CK^+^ patients with the identified phenotypes of (A) PD-L1 (*p* < 0.001 and *p* = 0.008 among the phenotypes at baseline and EOT, respectively) and (B) pS6 (*p* = 0.004 and *p* = 0.005 among the phenotypes at baseline and EOT, respectively). B (white bars), baseline; P 1st (purple bars), post 1st cycle; EOT (green bars), end of treatment. Figure S5: *pS6* gene expression and clinical outcome. Correlation of *pS6* expression with (A) first progression survival and (B) OS in NSCLC patients using KMplot (https://kmplot.com/analysis, accessed on 3 March 2022).

## Abbreviations

B, baseline; CK, cytokeratin; CTCs, circulating tumor cells; DFS, disease-free survival; EGFR, epidermal growth factor receptor; EMT, epithelial-to-mesenchymal transition; EOT, end of treatment; FBS, fetal bovine serum; ICIs, immune checkpoint inhibitors; NSCLC, non-small cell lung cancer; OS, overall survival; P 1st, post 1st cycle; PBMCs, peripheral blood mononuclear cells; PD-1, programmed cell death protein-1; PD-L1, programmed cell death ligand-1; PFS, progression-free survival; pS6, phospho-S6 ribosomal protein; TKI, tyrosine kinase inhibitor.
