# Supplementary material for: PD-L1/pS6 in Circulating Tumor Cells (CTCs) during Osimertinib Treatment in Patients with Non-Small Cell Lung Cancer (NSCLC)
Source: Biomedicines. 2022 Aug 5;10(8):1893. doi: 10.3390/biomedicines10081893 (PMC9405335; doi:10.3390/biomedicines10081893)
Supplement: Supplementary file 1 [file biomedicines-10-01893-s001.zip › biomedicines-1804527-supplementary.pdf]

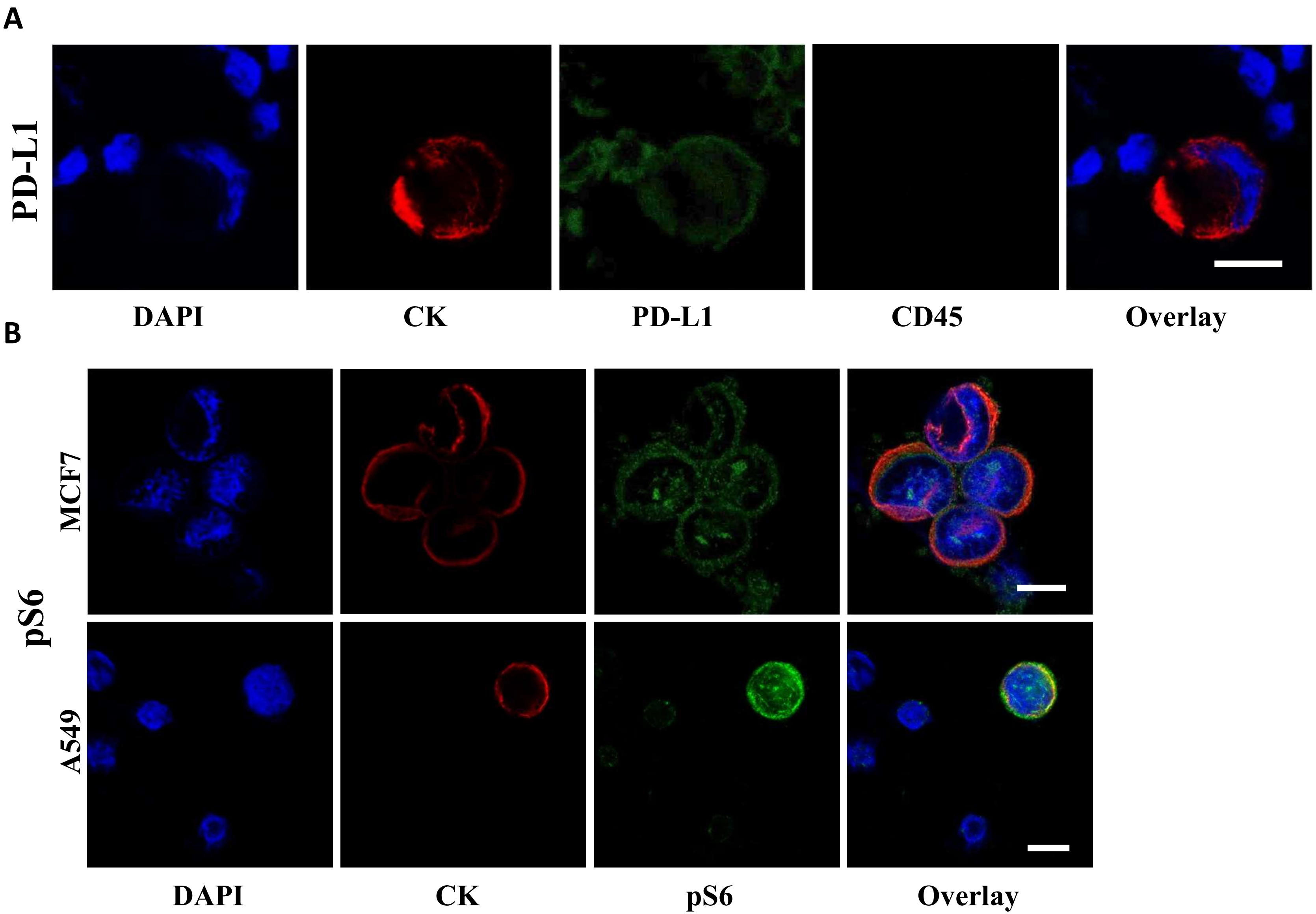

Figure S1. Cytospins of PBMCs from normal donors spiked with (A) H1299 and (B) MCF7 (breast) or A549 (lung) cells and stained for (A) CK (red)/PD-L1 (green)/CD45 (grey) and (B) CK (red)/pS6 (green). Overlays are presented. Scale bars = 10 μm.

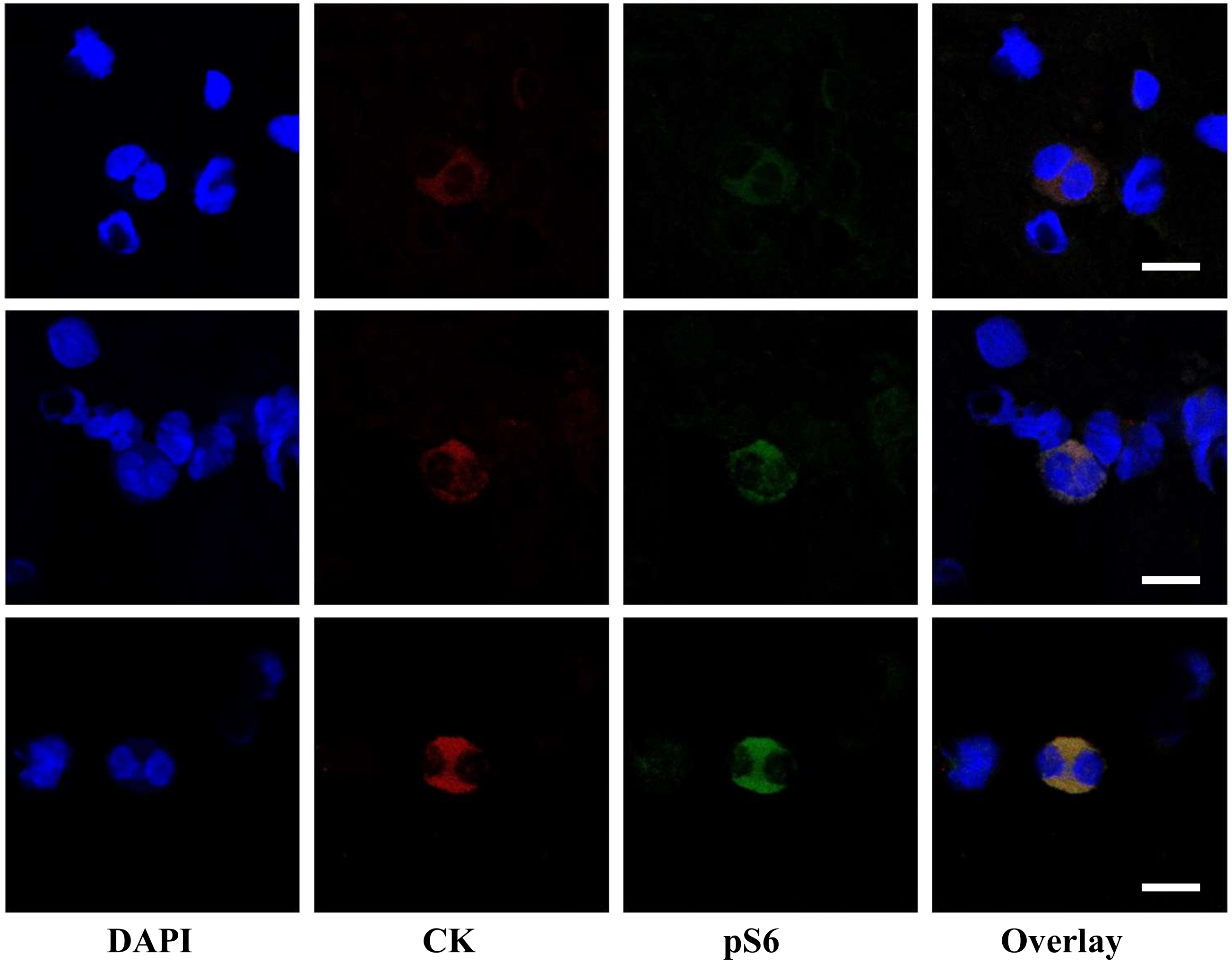

Figure S2. Representative fields with multiple cells. CTCs are stained for CK (red) and pS6 (green), and nuclei (blue) are visible with DAPI. The overlay of all images is also presented. Scale bars = 10 μm.

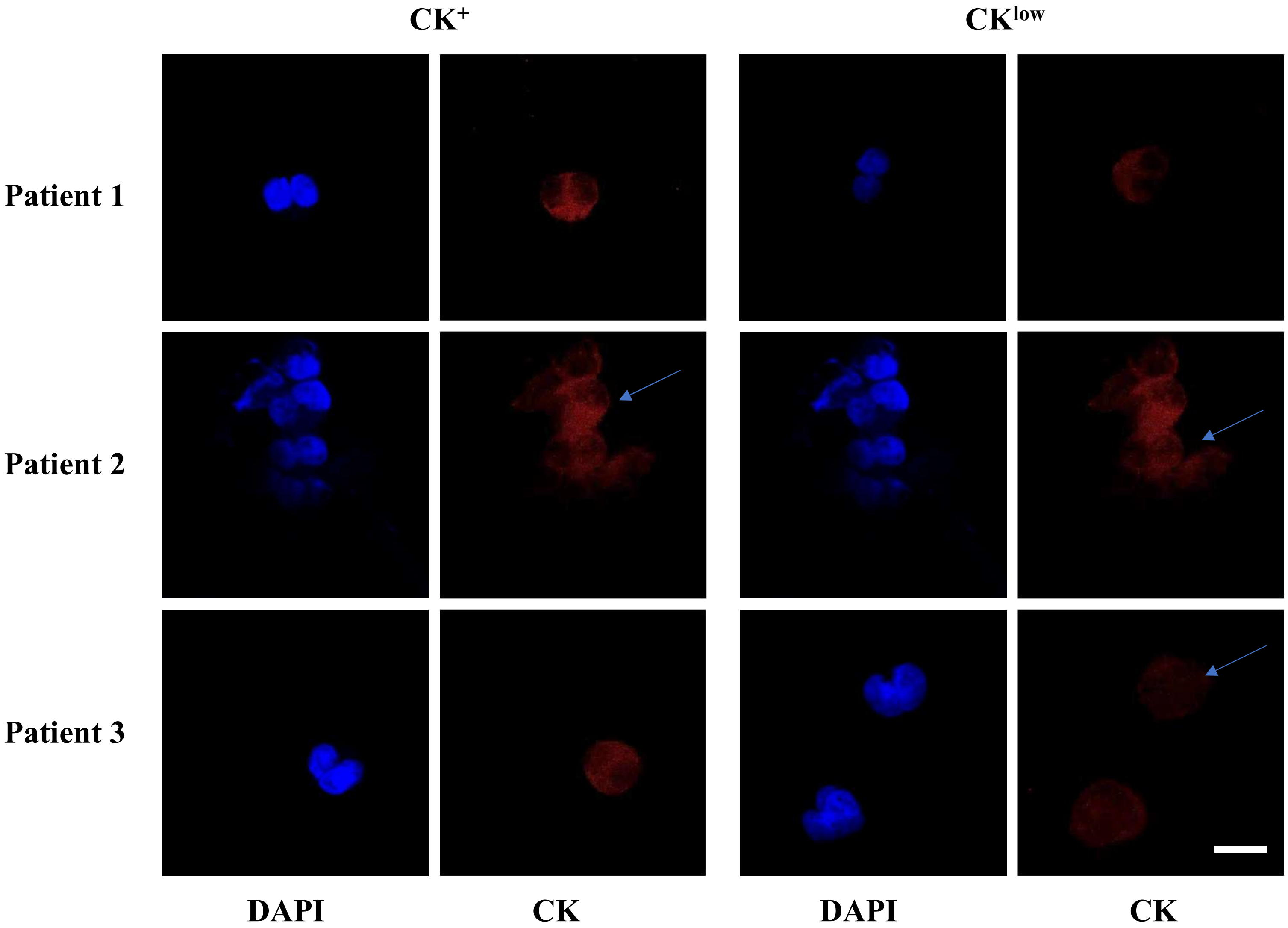

Figure S3. Representative panels of CK+- and CKlow-expressing cells among the same patients. CTCs are stained for CK (red), and nuclei (blue) are visible with DAPI. Arrows pinpoint the cell of interest. Scale bar = 10 μm.

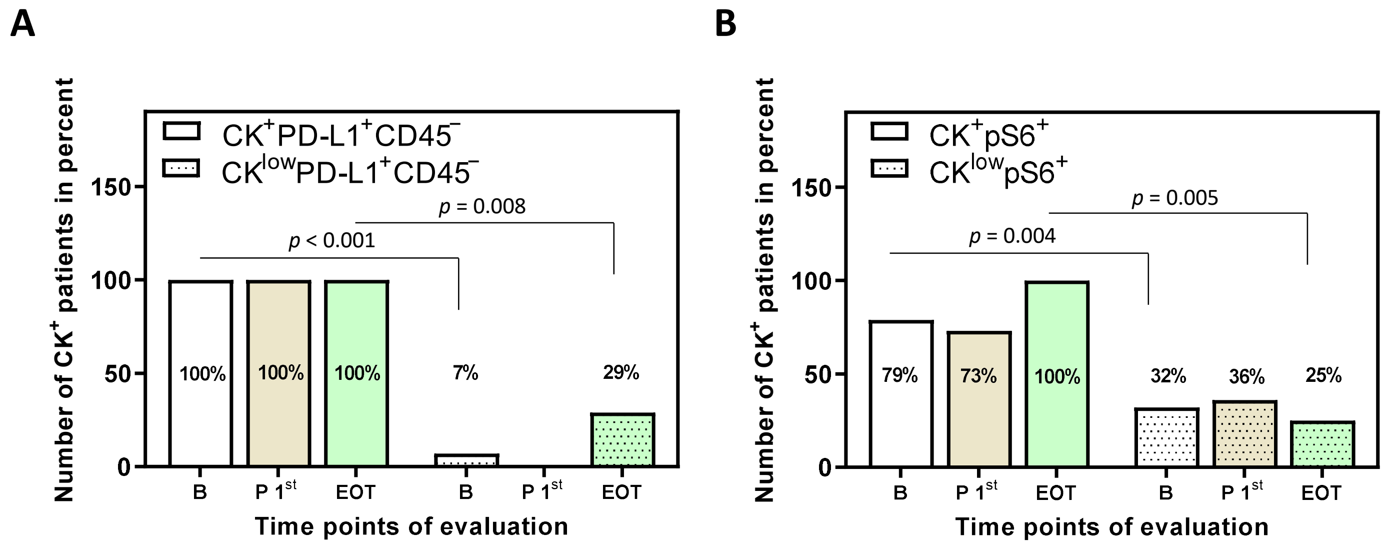

Figure S4. Phenotypes of PD-L1 and pS6 identified in NSCLC patients. Percentage of CK + patients with the identified phenotypes of (A) PD-L1 ($p < 0.001$ and $p = 0.008$ among the phenotypes at baseline and EOT, respectively) and (B) pS6 ($p = 0.004$ and $p = 0.005$ among the phenotypes at baseline and EOT, respectively). B (white bars), baseline; P 1st (purple bars), post 1st cycle; EOT (green bars), end of treatment.

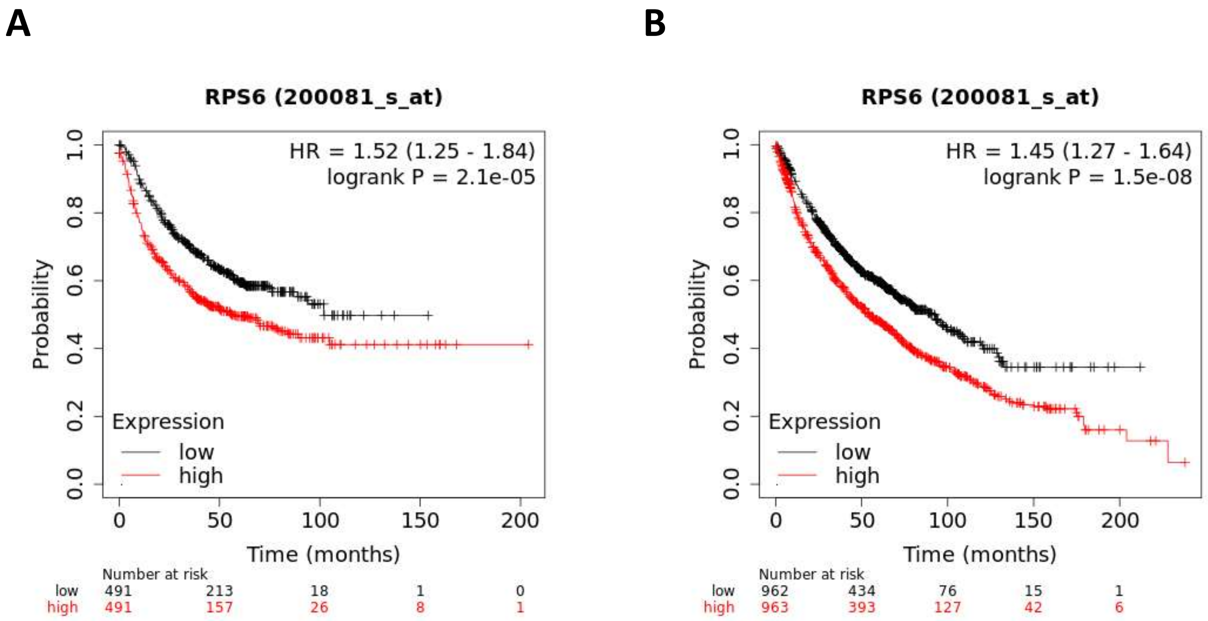

Figure S5. pS6 gene expression and clinical outcome. Correlation of pS6 expression with (A) first progression survival and (B) OS in NSCLC patients using KMplot (https://kmplot.com/analysis, accessed on 3 March 2022).
